# Bone Density and Cortical Thickness in Normal, Osteopenic, and Osteoporotic Sacra

**DOI:** 10.4061/2010/504078

**Published:** 2010-06-09

**Authors:** Andrew M. Richards, Nathan W. Coleman, Trevor A. Knight, Stephen M. Belkoff, Simon C. Mears

**Affiliations:** International Center for Orthopaedic Advancement, Department of Orthopaedic Surgery, The Johns Hopkins Bayview Medical Center, 3rd Floor, Alpha Center, 5210 Eastern Avenue, Baltimore, MD 21224-2780, USA

## Abstract

It is unclear if a decrease in cancellous bone density or cortical bone thickness is related to sacral insufficiency fractures. We hypothesized that reduction in overall bone density leads to local reductions in bone density and cortical thickness in cadaveric sacra that match clinically observed fracture patterns in patients with sacral insufficiency fractures. We used quantitative computed tomography to measure cancellous density and cortical thickness in multiple areas of normal, osteopenic, and osteoporotic sacra. Cancellous bone density was significantly lower in osteoporotic specimens in the central and anterior regions of the sacral ala compared with other regions of these specimens. Cortical thickness decreased uniformly in all regions of osteopenic and osteoporotic specimens. These results support our hypothesis that areas of the sacrum where sacral insufficiency fractures often occur have significantly larger decreases in cancellous bone density; however, they do not support the hypothesis that these areas have local reduction of cortical bone thickness.

## 1. Introduction

Pelvic fractures in the elderly usually occur from a low-energy fall onto the side, which most often produces a pubic ramus fracture in combination with a cortical disruption to the anterior aspect of the sacrum [1], or from repetitive axial stresses, which may cause a sacral insufficiency fracture (SIF) through the weakened bone of the sacrum. The typical SIF pattern is a longitudinal fracture extending parallel to the sacroiliac joint lateral to the sacral foramina [2] that may involve both sides of the sacrum or have a horizontal component. It is unknown if SIFs are caused by regional variations in cancellous bone density or cortical bone thickness.

In the current study, we measured cancellous bone density and cortical thickness in normal, osteopenic, and osteoporotic sacra to determine if changes in density or cortical thickness were associated with the clinically observed patterns of SIF.

## 2. Materials and Methods

Human cadaveric pelves were obtained from the Maryland State Anatomy Board, stripped of soft tissue, and inspected visually and radiographically. Specimens with previous surgery, fractures, or pathologic changes were excluded. Thirty-four pelves underwent bone density measurement via dual-energy X-ray absorptiometry scanning (Discovery QDR DEXA Scanner, Hologic, Inc., Bedford, MA) at L1-L4. Bone density measurement of the lumbar spine has been shown to correlate consistently with overall bone density of the S1 vertebrae [3]. According to the World Health Organization definitions [4], 11 pelves were normal (*t*-score>−1), 12 were osteopenic (*t*-score between −1 and −2.5), and 11 were osteoporotic (*t*-score <−2.5) (Table 1).

After dual-energy X-ray absorptiometry scanning, the specimens were double-wrapped in plastic bags and stored at −20°C. The specimens were thawed at room temperature (20°C) for 24 hours before testing. Computed tomography (CT) scans were obtained with an Aquilion 16 multidetector CT scanner (Toshiba America Medical Systems, Tustin, CA). The pelves were scanned in the prone position on a wedge with the cranial end tilted up by 30°. The CT gantry was positioned parallel to the upper endplate of the S1 vertebra to capture true axial slices of the S1 vertebra. Sacral positioning was confirmed with a scout acquisition series, and pelvic position was adjusted according to the scout until correct. A CT bone density phantom (Computerized Imaging Referencing Systems, Inc., Norfolk, VA) was included in each 1-mm thick slice, and the slices were saved to disc. Five images of each S1 vertebra were analyzed: 1 immediately distal to the upper endplate; 1 immediately proximal to the lower endplate; 1 central to those first 2 images; and 2 slices, each of which was equidistant between an endplate and a central slice.

**Figure 1:**
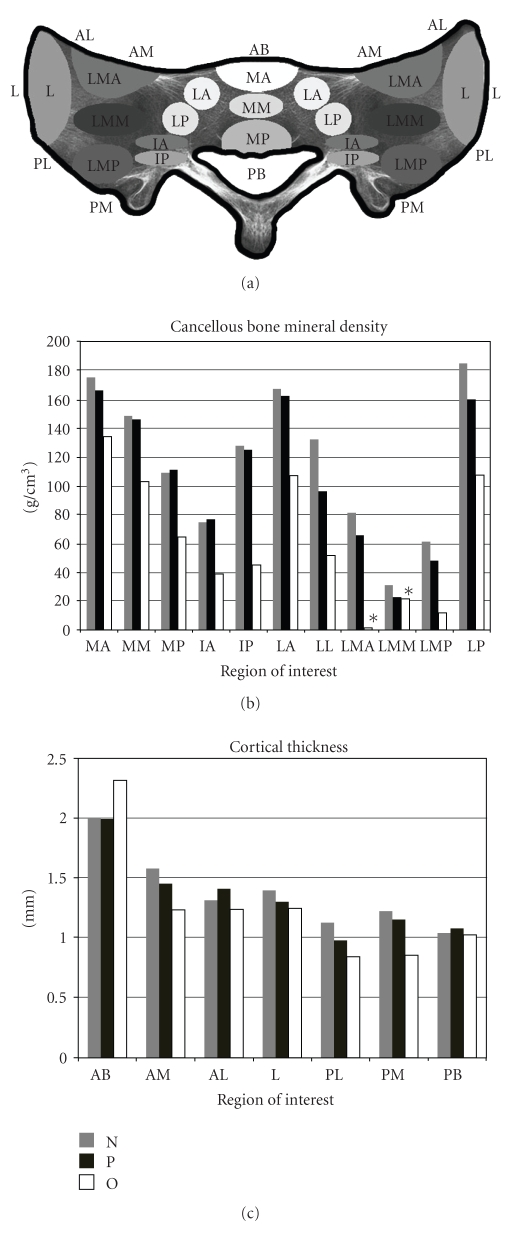
Cancellous and cortical regions of interest with cancellous bone density and cortical thickness. (a) Letter outside the sacrum represents the location of cortical measurements on each slice; the inside letters and ovals represent the cancellous regions of interest. (b) Bone mineral density by region of interest for the normal, osteopenic, and osteoporotic groups. (c) Cortical thickness by region of interest for the normal, osteopenic, and osteoporotic groups. ∗ negative values (see Table 2).

The scans were analyzed with Vitrea Imaging Software (Vital Images, Inc., Minnetonka, MN), which allows definition of discrete areas of the image. The mean Hounsfield unit was calculated for 19 specific areas for each axial slice, as defined by Zheng et al. [5] (Figure 1). The placement of the areas for measurement was agreed on by 2 observers (A.M.R. and N.W.C.) in each case.

For each slice of each sacrum, the density of the hydroxyapatite reference rods within the CT phantom was measured in Hounsfield units. To calculate the actual bone mineral density (BMD, in g/cm^3^) of the cancellous bone of the sacrum, we used linear regression analysis to convert the observed Hounsfield unit for each defined area to BMD by referencing it against the known density of the hydroxyapatite phantom. Because we found no significant differences (*P* < .05) between left and right cancellous bone density or cortical thickness, density and thickness measurements from right and left sides were averaged.

Cortical bone thickness measurements were also obtained in 18 of the 34 pelves. Of those 18 pelves, 6 were normal, 6 were osteopenic, and 6 were osteoporotic.

Cortical thickness was measured using the measurement tool of the Vitrea Imaging Software (Vital Images, Inc.). Twelve regions of interest of the sacral cortex were identified (Figure 1): 2 in the sacral body (1 anterior and 1 posterior) and 10 in the cortex of the sacral ala (5 symmetrical regions on each side: 2 anterior, 2 posterior, and 1 lateral). The identification of the regions of interest for density and thickness measurements was agreed on by 2 observers (A.M.R. and N.W.C.) in each case.

We tested for significant (*P* < .05) effects of location and sacral nominal density (normal, osteopenic, or osteoporotic) on cancellous density or cortical thickness using multiple linear regressions accounting for random effects (Stata 10, StataCorp LP, College Station, TX).

## 3. Results

### 3.1. Cancellous Bone Density

Cancellous bone density was significantly lower in the osteoporotic group than in the osteopenic or normal groups (Figure 1). Cancellous bone density varied significantly as a function of location, with the most decrease in density in the areas lateral to the neural foramina compared with areas more medial or lateral (Table 2).

### 3.2. Cortical Bone Thickness

Controlling for location, cortical thickness in osteoporotic sacra was significantly less than that in normal and osteopenic sacra.The anterior cortex of the body of the sacrum was significantly thicker than the other cortical locations of interest in all 3 categories of sacra. Cortical thickness was greater in the anterior regions than in the posterior regions. Cortical thickness was not significantly different between normal and osteopenic sacra when controlling for location (Table 3).

## 4. Discussion

The results of this study support the hypothesis that specific areas of the sacrum have greater loss of cancellous bone density in osteoporotic bone than in bone with normal density or osteopenia. The central and anterior parts of the sacral ala (where SIFs often occur) had significantly more loss in trabecular bone density than did other sacral regions. The decreased trabecular bone density of this central area of the sacral ala corresponds to the “fatty sphere” or “ala void” first described by de Peretti et al. [6]. This void is a potentially weak area in the structure of the osteoporotic sacrum, and SIFs occur through this area of the sacrum lateral to the foramen. These fractures can be bilateral and have horizontal extensions [2]. The area of decreased cancellous bone density in osteoporotic specimens coincides with the typical location of SIFs described clinically [7, 8] and in a cadaveric biomechanical study [9].

The results of our study do not support the hypothesis that specific areas of the sacrum have decreases in cortical thickness in osteoporotic specimens compared with normal or osteopenic specimens. In the osteoporotic pelvis, the anterior cortex lateral to the neural foramina is the area in which SIFs occur [10–12]. However, in our study, this region did not have the thinnest cortex in osteoporotic specimens compared with other regions of the sacrum. Although there was generalized cortical thinning with increasing osteoporosis, no specific area had a greater loss of cortical bone. Ebraheim et al. [3, 13] and Peretz et al. [14] have measured cortical thickness in the sacrum; however, neither group made an assessment of overall bone density of the specimens to define the degree of porosity of their specimens. Ebraheim et al. [3, 13] performed quantitative CT on 40 sacra (donor age, 61 to 67 years) and showed that the average anterior cortical thickness was 2.5 ± 0.6 mm. Peretz et al. [14] measured cortical thickness with direct microscopic visualization. In a group of 17 specimens, they found a range of cortical thickness between 0.5 and 2.25 mm, and an average thickness of slightly more than 1.28 mm. In our study, the mean cortical thickness of normal specimens was 1.36 mm, which closely correlates with the thickness found by Peretz et al. [14], and is markedly less than that found by Ebraheim et al. [3, 13].

There are weaknesses in our study. We did not use a specific quantitative CT machine. The CT machine that we did use, however, was calibrated each day and the density measurement was controlled by the use of a CT bone density phantom. Mazess [15] has shown that the high fat content of cancellous bone, especially at low bone mineral densities, produces an error of up to 10% when measuring BMD. The presence of yellow marrow (high fat content) causes an underestimation of cancellous BMD. The very low measurements of BMD found in the osteoporotic group should be seen as an underestimate of the true value.

## 5. Conclusions

Our results show that the cancellous BMD in the areas just lateral to the neural foramina is greatly reduced in the osteoporotic sacrum. The anterior cortex of the sacral ala does not undergo excessive thinning when compared with other regions of the sacrum as specimens become osteoporotic. These areas of greatest cancellous bone loss correspond to the location of SIFs. Therefore, cancellous strength may be more important than cortical thickness with SIFs. Additional work is required to further define the role of cortical versus cancellous bone strength with different force mechanisms.

## Figures and Tables

**Table 1:** Demographics of specimens.

Specimen	Number	*t*-score		Donor Age		Male : Female ratio
		Mean	Range	Mean	Range	
Cancellous bone measurement						
Normal	11	0.008	1.99 to −0.91	80.4	46 to 90	6 : 5
Osteopenic	12	−1.66	−1.05 to −2.44	77.6	50 to 90	7 : 5
Osteoporotic	11	−4.175	−2.79 to −7.09	81.1	65 to 97	3 : 8

Cortical thickness analysis						
Normal	6	−0.237	0.7 to −0.86	82.4	62 to 90	4 : 2
Osteopenic	6	−1.49	−1.05 to −2.16	79.6	63 to 86	3 : 3
Osteoporotic	6	−3.75	−2.9 to −4.74	81.5	70 to 86	2 : 4

**Table 2:** Cancellous bone mineral density by region and condition.

Location*	BMD (g/cm^3^)	95% Confidence Interval	
		Lower	Upper
IA			
N	75.12	52.98	97.26
P	77.05	49.03	105.08
O	39.15	5.33	72.97

IP			
N	128.14	105.51	150.77
P	125.43	92.66	158.21
O	45.42	26.30	64.55

LA			
N	167.86	147.92	187.80
P	163.05	139.15	186.95
O	107.59	90.18	125.01

LL			
N	132.62	108.53	156.71
P	96.43	75.39	117.48
O	51.76	39.52	63.99

LMA			
N	81.76	56.95	106.57
P	65.95	40.48	91.43
O	−1.33	−12.35	9.70

LMM			
N	31.33	14.94	47.72
P	22.83	5.92	39.74
O	−21.46	−27.41	−15.52

LMP			
N	61.46	44.79	78.14
P	48.17	30.65	65.68
O	11.70	−0.46	23.86

LP			
N	185.79	156.93	214.65
P	160.53	131.15	189.90
O	107.89	94.39	121.40

MA			
N	175.86	140.73	210.99
P	167.00	134.38	199.62
O	134.71	99.50	169.93

MM			
N	149.30	120.27	178.33
P	146.83	110.41	183.26
O	103.47	74.63	132.31

MP			
N	109.60	79.33	139.87
P	111.57	76.69	146.44
O	64.86	42.51	87.21

BMD: body mass index; N: normal; O: osteoporotic; P: osteopenic.

*For definition of locations, please see Figure 1.

**Table 3:** Cortical thickness by region and condition.

Location*	Cortical thickness	95% Confidence interval	
	(mm)	Lower	Upper
AB			
N	2.01	1.76	2.25
P	2.00	1.69	2.30
O	2.32	2.04	2.60

AL			
N	1.32	1.22	1.41
P	1.41	1.30	1.52
O	1.24	1.16	1.32

L			
N	1.40	1.30	1.50
P	1.31	1.24	1.37
O	1.25	1.14	1.36

PB			
N	1.04	0.97	1.12
P	1.08	0.98	1.18
O	1.03	0.95	1.11

PL			
N	1.13	1.03	1.23
P	0.98	0.91	1.06
O	0.85	0.79	0.90

PM			
N	1.23	1.14	1.32
P	1.15	1.08	1.23
O	0.86	0.79	0.92

AM			
N	1.58	1.46	1.70
P	1.46	1.31	1.60
O	1.24	1.18	1.30

BMD: body mass index; N: normal; O: osteoporotic; P: osteopenic.

*For definition of locations, please see Figure 1.
